# Sustainable Strategies for Converting Organic, Electronic, and Plastic Waste From Municipal Solid Waste Into Functional Materials

**DOI:** 10.1002/gch2.202400240

**Published:** 2025-03-07

**Authors:** Abdelaziz Gouda, Nour Merhi, Mohamad Hmadeh, Teresa Cecchi, Clara Santato, Mohini Sain

**Affiliations:** ^1^ Department of Applied Chemistry and Chemical Engineering University of Toronto 80 St. George Street Toronto ON M5S 3H6 Canada; ^2^ Centre for Biocomposites and Biomaterials Processing Division of Forestry Daniels Faculty of Architecture Landscape and Design University of Toronto Toronto ON M5S 3E8 Canada; ^3^ Department of Mechanical and Industrial Engineering University of Toronto Toronto ON M5S 3G8 Canada; ^4^ Department of Chemistry American University of Beirut Riad El‐Solh, P.O. Box 11‐0236 Beirut Lebanon; ^5^ Istituto Tecnico Tecnologico (ITT) G. and M. Montani Fermo 63900 Italy; ^6^ Engineering Physics Polytechnique Montreal Montreal QC H3T 1J4 Canada

**Keywords:** municipal solid waste, thermochemical valorization, solar energy, metal–organic framework, green hydrometallurgy, optoelectronics

## Abstract

The valorization of municipal solid waste permits to obtain sustainable functional materials. As the urban population burgeons, so does the volume of discarded waste, presenting both a challenge and an opportunity. Harnessing the materials and the latent energy within this solid waste not only addresses the issue of disposal but also contributes to the innovation of functional materials with applications in the energy, electronics, and environment sectors. In this perspective, technologies for converting, after sorting, municipal solid waste into valuable metals, chemicals, and fuels are critically analyzed. Innovative approaches to convert organic waste into functional carbon materials and to create, from plastic and electronic wastes, metal–organic frameworks for energy conversion, storage, and CO_2_ adsorption and conversion are proposed. Green hydrometallurgy routes that permit the recovery of precious metals avoiding noble metals’ oxidative leaching, thus avoiding their downcycling, are also highlighted. The reclaimed precious metals hold promise for use in optoelectronic devices.

## Graphical abstract

This Perspective highlights sustainable strategies to convert municipal solid waste into functional materials. It discusses innovative methods to transform organic waste into carbon materials, plastic and metal wastes into metal–organic frameworks. It includes precious metals' recovery using green hydrometallurgy. These approaches address waste disposal challenges while enabling energy, electronics, and environmental applications.

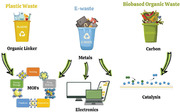

## 1 Introduction

*“We Do Not Inherit the Earth from Our Ancestors; We Borrow It from Our Children”* ^[^ ^1^ ^]^

**Figure 1:**
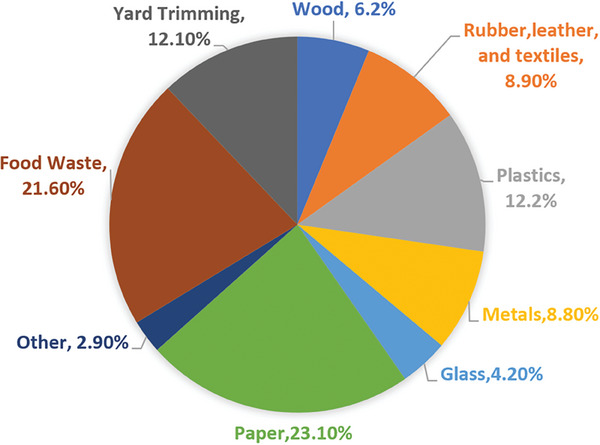
MSW composition in the U.S. Adapted from ^[^ ^2^ ^]^2018, U.S. Environmental Protection Agency.

Solid Municipal Waste (SMW), or Municipal Solid Waste (MSW), presents a significant environmental challenge in terms of landfill congestion and greenhouse gas emissions. MSW encompasses everyday discarded items from households, businesses, and institutions, including residential waste (food scraps, plastics, paper), commercial waste (packaging, e‐waste), yard debris, and occasionally construction materials (**Figure** **1**).

**Figure 2:**
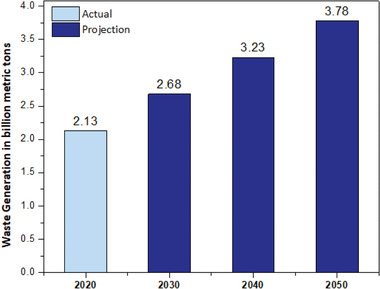
The global generation of MSW in 2016 with projections for 2030 and 2050, indicating an estimated increase to 3.4 billion metric tons by 2050^[^ ^3^ ^]^ 2023, Statista.

Current and projected data on the generation of waste at the global level highlight the urgent need for enhanced waste management practices and sustainable solutions for MSW (**Figure** **2**).

After being collected and sorted, waste is transported to various sites for landfilling, recycling, composting, or recovery. Landfilling remains the most used method of waste disposal and treatment worldwide. Sudokwon, located in South Korea, is one of the largest landfills in the world, receiving ≈20 000 tons of waste per day.^[^ ^4^ ^]^ Landfilling contributes significantly to greenhouse gas (GHG) emissions and exacerbates space constraints due to its reliance on large, finite land areas.

**Organic waste** represents a significant component of MSW (for example: 75 million tons, or 34% of the total MSW generated annually across the European Union in 2019^[^ ^5^ ^]^), and poses substantial environmental and management challenges due to its volume, decomposition characteristics, and potential for GHGs. Organic waste, which includes food scraps, yard debris, biodegradable materials, plant or animal‐derived residues, and sewage sludge, represents a diverse range of biological materials with significant potential for valorization. Despite its potential, 69% of this waste is managed through incineration or landfilling, leaving it underutilized. By setting a target of 65% for the reuse and recycling of MSW by 2030, the European Commission is encouraging innovative solutions. Improper disposal, such as landfilling or open dumping, leads to the release of methane—a potent GHG—during anaerobic decomposition, contributing to climate change as landfilled organic waste contributes 20% of global methane emissions.^[^ ^6^ ^]^ Methane is far more threatening at trapping heat than CO₂. Over a 20‐year period, methane's global warming potential (GWP) is ≈84–87 times greater than that of CO₂, and over a 100‐year period, it is ≈28–34 times greater.^[^ ^7^ ^]^ Notably, food waste, amounting to 1.3 billion tons annually, ranks as the third‐largest source of GHG emissions globally, accounting for about 6% of total emissions.^[^ ^6^, ^8^ ^]^ Additionally, the leachates from organic waste can contaminate soil and groundwater, exacerbating environmental concerns.

The efficient management and valorization of organic waste are essential for addressing these challenges. Traditional disposal methods, like incineration or landfilling, often squander the resource potential of organic waste and create further environmental burdens.^[^ ^9^ ^]^ In contrast, sustainable valorization methods aim to transform organic waste into valuable products through carbonization and/or activation processes, such as biofuels, biochemicals, compost, or functional carbon materials for potential energy storage and catalysis applications.^[^ ^10^ ^]^

Waste valorization focuses on transforming waste into valuable resources by processing residues or byproducts into raw materials, reusing discarded products as inputs or energy sources, integrating waste into manufacturing processes, or incorporating it into finished products.^[^ ^11^ ^]^ Recycling, one of the most common valorization methods, involves recovering and reprocessing waste into new products, often resulting in lower‐quality materials, a process known as downcycling.^[^ ^12^ ^]^ In contrast, upcycling enhances waste into higher‐value, higher‐functionality products, making it the preferred approach for sustainable waste valorization.^[^ ^13^ ^]^

Despite its potential, organic waste valorization faces several challenges, including the heterogeneous nature of waste streams, contamination with non‐biodegradable materials, and the need for advanced processing technologies. Economic and logistical barriers, such as high collection, transportation, and infrastructure costs, further hinder widespread adoption. Overcoming these obstacles through innovative, scalable, and cost‐effective strategies is essential for advancing a circular economy and sustainable waste management systems.^[^ ^9^ ^]^ One promising approach involves converting sorted organic waste into functional carbon materials using various valorization strategies. In this Perspective, we will critically discuss these techniques in terms of their versatility, sustainability, energy requirements, and the critical properties of the resulting products for different potential energy storage and catalysis applications, boosting the transition toward a greener and more circular economy.

**Plastic waste**, a second major component of MSW, particularly discarded plastic bottles, poses a significant environmental challenge due to its widespread use and non‐biodegradable nature.^[^ ^14^ ^]^ Plastic materials, especially polyethylene terephthalate (PET), are widely used in food packaging, water and beverage containers, and other everyday uses due to their lightweight, low cost, chemical inertness, and other properties. However, the properties that make PET advantageous for use in everyday items also contribute to its environmental persistence. PET's inability to decompose, combined with the sheer volume of plastic waste generated, contributes to a buildup in the environment, resulting in alarming environmental consequences.^[^ ^15^ ^]^ If plastic production and waste management processes remain unchanged, approximately 12,000 million metric tons of plastic waste could accumulate in landfills or the natural environment by 2050.^[^ ^16^ ^]^ Inefficient disposal of plastic waste and uncontrolled recycling methods could significantly contribute to environmental pollution. Under an ambitious scenario, plastic emissions to aquatic ecosystems are projected to range between 20 and 53 million tons per year by 2030.^[^ ^17^ ^]^ These emissions, along with the degradation and mismanagement of plastic waste, such as landfilling and incineration may further drive greenhouse gas emissions, including carbon dioxide (CO_2_) and methane (CH_4_), intensifying climate change. Microplastics pollution due to the weathering of plastic items is a global issue and the leaching of toxic compounds (e.g., flame retardants) from plastic litter poses additional environmental concerns.^[^ ^18^ ^]^

Recycled PET products have limited value, making the recycling process less attractive compared to the cost‐effectiveness of using new PET. Conventional recycling methods, such as mechanical,^[^ ^19^ ^]^ thermal,^[^ ^20^ ^]^ and chemical,^[^ ^21^ ^]^ raise environmental concerns as they can be energy‐intensive or produce secondary pollutants.^[^ ^22^ ^]^ These challenges hinder the economic and environmental feasibility of PET recycling compared to using virgin materials. In addition to the conventional recycling processes, two concepts attracting increasing research attention have emerged: closed‐loop recycling and upcycling.

Closed‐loop recycling refers to a process in which recovered materials are directly recycled back into the same production cycle, maintaining their useful properties across multiple recycling loops. For instance, recent innovations include the development of new polymeric materials like polyester^[^ ^23^ ^]^ and polyolefin‐like materials,^[^ ^24^ ^]^ which can be efficiently recycled into their original monomers. Chemical upcycling, where plastic waste is converted into valuable chemicals and materials, holds promise in addressing both environmental and economic issues related to plastic waste. One promising method involves converting PET into high‐value materials, such as metal‐ organic frameworks, a process that will be explored in detail in this study.

**Metal–organic frameworks (MOFs)**, a unique class of porous hybrid materials, have gained significant attention as valuable outcomes of recycling efforts, particularly from waste. Their synthesis from non‐biodegradable plastics, such as PET, represents an approach to addressing environmental challenges. Unlike conventional recycling methods that often involve high energy consumption, economic limitations, and secondary pollution, the production of MOFs from waste provides a practical and environmentally sustainable alternative. PET plastics, composed of ≈85% terephthalic acid (1,4‐benzendicarboxylic acid, BDC) monomer,^[^ ^25^ ^]^ a crucial industrial raw material, are particularly advantageous for MOF synthesis. BDC is a widely used organic linker in the preparation of various MOFs.^[^ ^26^ ^]^ The process of transforming waste PET into MOFs not only mitigates plastic pollution but also results in advanced materials with diverse applications. In addition, to achieve the practical implementation of the waste‐to‐MOF approach, it is crucial to establish a comprehensive synthetic process that utilizes waste sources for both the organic linker and the metal centre. This would make MOF synthesis more sustainable and cost‐efficient, paving the way for large‐scale production. This innovative use of waste aligns with the principles of a circular economy by converting pollutants into high‐value materials. MOFs derived from waste exhibit exceptional properties, enabling their application in areas such as catalysis, energy storage, and drug delivery. Their production highlights the potential of integrating waste management with advanced material development, establishing MOFs as a significant and sustainable resource in addressing global environmental concerns.

**Electrical and electronic equipment (EEE)** encompasses all products with circuitry or electrical components powered by a battery or electricity, becoming waste EEE (WEEE or e‐waste) when discarded by its owner without intent for reuse.^[^ ^27^ ^]^ E‐waste constitutes over 5% of total MSW worldwide. E‐waste generation is projected to grow by 3–5% annually, nearly three times the yearly growth rate of MSW.^[^ ^27^ ^]^ The amount of e‐waste reached 62 million metric tons in 2022, expected to rise another 32%, to 82 million tonnes in 2030.^[^ ^27^ ^]^ Discarded phones, tablets, laptops, and other gadgets have an estimated annual value of $62.5 billion, containing 100 times more gold per unit of mass than gold ore. However, only about 20% of the world's e‐waste is recycled. Recycling e‐waste is crucial for sustainability, aligning with the United Nations' definition of sustainability, i.e., “meeting the needs of the present without compromising the ability of future generations to meet their own needs”. This practice helps mitigate the pressure of extracting finite resources and reduces the risk of geopolitical conflicts over mineral extraction.

**44 critical elements** of the 118 elements that make up everything, will face supply limitations in the coming years, including rare earth elements and precious metals.^[^ ^28^ ^]^ The Energy Act of 2020 in the United States defines a “critical material” as any non‐fuel mineral, element, substance, or material that the Secretary of Energy determines has a high risk of supply chain disruption and serves an essential function in one or more energy technologies.^[^ ^30^ ^]^ Similarly, a “critical mineral” is any mineral, element, substance, or material designated as critical by the US Secretary of the Interior. The 2022 final list of critical minerals included 50 minerals, such as antimony, bismuth, and titanium. The 2023 Critical Materials List issued by the US Department of Energy includes key materials for energy, such as aluminium, cobalt, copper, and lithium, among others.

**Figure 3:**
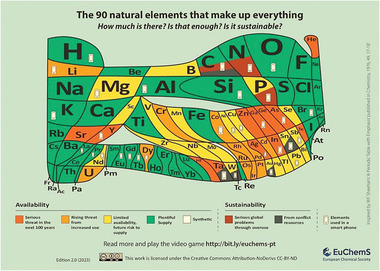
Periodic Table – 2.0 Adapted from^[^ ^29^ ^]^ 2023, EuChemS.

In 2019, the International Year of the Periodic Table (IYPT2019), EuChemS, the European Chemical Society, proposed a version of the Periodic Table to emphasize the theme of critical (*endangered*) elements, urging reflection and action on the issue (**Figure** **3**).

**Urban mining** extracts resources from complex waste streams, offering significant economic opportunities, especially in countries often regarded as e‐waste dumping sites.^[^ ^31^, ^32^ ^]^

**In this context, green hydrometallurgy** emerges as a sustainable and innovative approach to address these challenges. This method involves using environmentally friendly chemical processes, such as bio‐based solvents and mild reaction conditions, to extract valuable precious metals like gold, silver, and palladium from e‐waste.^[^ ^33^ ^]^ Unlike traditional methods that often rely on energy‐intensive smelting or the use of hazardous chemicals, green hydrometallurgy minimizes environmental pollution and health risks while achieving high recovery efficiencies.^[^ ^34^ ^]^

By integrating green hydrometallurgy into e‐waste management systems, it is possible to create a circular economy that not only reduces environmental and health hazards but also contributes to sustainable advancements in energy and catalysis technologies.

**The relevance of this perspective** is to highlight the potential of leveraging materials derived from sorted MSW to create functional materials with diverse applications. We detail the conversion of organic waste into functional carbon materials for potential energy storage and catalysis applications. Additionally, we explore the utilization of plastics and metals from e‐waste in the fabrication of metal–organic frameworks (MOFs) and their further application in sustainable technologies. Beyond MOFs, we discuss environmentally friendly hydrometallurgical methods for recovering precious metals while preventing downcycling and examine the valorization of recycled metals for optoelectronic applications. By transforming waste into economically valuable and functional resources, this work contributes to advancing sustainable technologies, minimizing environmental impact, and promoting broader societal and health benefits.

## 2 Organic Waste Treatment Processes

The valorization of organic waste focuses on converting it into value‐added materials and chemical fuels through diverse processes, including biochemical techniques based on anaerobic digestion, fermentation, or photobiological hydrogen production and thermo‐chemical conversion.^[^ ^35^ ^]^ Among thermo‐chemical processes, gasification, pyrolysis, dry torrefaction, hydrothermal carbonization, and ionothermal carbonization have been widely explored for their efficiency and versatility.^[^ ^36^ ^]^ The selection of an appropriate process depends on factors such as the feedstock's type and composition and the resulting products' intended applications, ranging from energy carriers to functional carbon materials. This adaptability makes organic waste valorization a critical pathway for sustainable resource utilization and circular economy development.

### 2.1 Biochemical Processes

#### 2.1.1 Anaerobic Digestion and Fermentation

Anaerobic digestion, a biological process where microorganisms break down organic matter in the absence of oxygen, yields biogas as a byproduct. This biogas, primarily composed of methane, can be used as a renewable energy source for electricity and heat generation. Organic waste thus becomes a bioenergy reservoir, contributing to a more sustainable and circular economy.^[^ ^37^ ^]^

Beyond biogas, the residual solid fraction from anaerobic digestion and other valorization processes can be further processed through appropriate thermochemical processes to produce bio‐based materials. These materials have shown promise in the development of functional energy materials. For example, biochar, a carbon‐rich material derived from the pyrolysis of organic waste, can be integrated into supercapacitors and batteries, offering a sustainable alternative to traditional carbon materials.^[^ ^35a–f^ ^]^

#### 2.1.2 Photobiological Hydrogen Production

Photobiological hydrogen production is a promising biochemical technique that utilizes light‐driven biological processes to produce hydrogen, leveraging microorganisms such as photosynthetic algae and cyanobacteria. These organisms, under anaerobic conditions and exposure to light, harness their photosystems to split water molecules, releasing hydrogen as a byproduct. Organic waste is an excellent substrate in this process, providing essential nutrients and carbon sources for microbial growth and metabolic activity. This method is particularly appealing due to its sustainability, as it uses renewable feedstocks and operates under mild conditions, minimizing energy inputs. Moreover, photobiological systems contribute to the dual benefit of waste valorization and clean hydrogen production, aligning with circular economy goals. Despite its potential, challenges such as low hydrogen yields, light penetration limitations, and sensitivity to oxygen inhibition need to be addressed through advancements in genetic engineering, reactor design, and optimization of culture conditions.^[^ ^38^ ^]^

### 2.2 Thermo‐Chemical Processes

#### 2.2.1 Gasification

Gasification is a thermo‐chemical process that converts carbonaceous materials (such as coal, biomass, and petroleum coke) into gaseous syngas, primarily composed of H_2_ and CO, and a low‐yield solid biochar, a form of charcoal employed to increase soil quality, and liquid tar and oil. This process occurs at high temperatures and under oxygen‐deficient conditions.^[^ ^36^, ^39^ ^]^

Biomass gasification involves four steps: drying, pyrolysis, partial combustion of gases, vapours, and char, and chemical reduction of the combustion products. Drying is done below 250 °C, pyrolysis at 250–500 °C, and the operating temperature of both partial combustion and reduction reaches 800–1200 °C. Air, oxygen, steam, or mixtures of these gases are commonly used as oxidizing agents.

Gasifying temperature, residence time, heating rate, equivalence ratio (ER) (actual oxidant‐fuel ratio to stoichiometric oxidant‐fuel ratio), and gasifying agent significantly influence the properties of the biochar from biomass gasification.^[^ ^40^ ^]^ A value of ER close to zero corresponds to pyrolysis, while a value equal to or greater than one indicates complete combustion. The gasifying temperature is the most important parameter affecting syngas components, and higher gasifying temperatures lead to reduced yields of biochar and tar, which is a byproduct obtained as a viscous liquid mixture of hydrocarbon compounds.

The usual product yields of this process are ≈85% syngas, 5% tar and oil, and 10% biochar.

Biomass gasification is similar to physical activation treatments for synthesizing activated carbons with high porosity when using oxygen, steam, or mixtures as gasifying agents.^[^ ^39a^ ^]^ However, biochar from gasification generally has lower porosity compared to biochar from pyrolysis processes, which is primarily due to the distinct conditions and steps involved in each technique.^[^ ^41^ ^]^ In gasification, the higher temperatures and limited oxygen supply lead to a more compact biochar with lower porosity. In contrast, pyrolysis, with its lower temperatures and oxygen‐free environment, allows for greater pore formation, resulting in a more porous biochar. Biochar is known for its applications in energy storage, conversion, and catalysis based on the physicochemical properties of biochar from biomass gasification.^[^ ^35a–f^ ^]^

#### 2.2.2 Pyrolysis

Pyrolysis operates at lower temperatures than incineration and occurs in the absence of oxygen, which limits the formation of toxic gases like dioxins and furans.^[^ ^42^ ^]^ Pyrolysis can be more energy‐efficient by converting waste into valuable byproducts like fuel, whereas incineration requires higher temperatures and generates pollutants. While landfilling is energy‐efficient short‐term, it causes long‐term environmental issues like methane emissions and land degradation. Under an inert atmosphere and with a wide operating temperature range of 300–900 °C, pyrolysis produces syngas, bio‐oil, and biochar, through the thermal decomposition of the organic waste.^[^ ^43^ ^]^ The heating rate, operating temperature, and residence time define the carbon properties. The pyrolysis conversion mechanism includes cracking, decarboxylation, hydrocracking, hydrodeoxygenation, and hydrogenation.^[^ ^44^ ^]^ Considering the operating conditions, pyrolysis can be classified into: slow, fast, flash, vacuum, microwave‐assisted, and solar thermal.^[^ ^40^ ^]^

**Slow Pyrolysis** is the preferred process for organic waste valorization to biochar. It is done at 350–900 °C and has a long residence time (≤1 h) and slow heating rate (5–7 °C min^−1^), yielding a relatively large amount of biochar (> 12 wt%) compared to bio‐oil and gaseous products.^[^ ^42^, ^45^ ^]^ The biochar produced from the slow pyrolysis is used as fertilizer, sorbents for wastewater, and soil remediation.^[^ ^46^ ^]^

**Fast Pyrolysis** yields up to 75 wt% bio‐oil, used as an energy carrier, and a yield of up to 12 wt% of biochar, the remainder being syngas.^[^ ^42^, ^47^ ^]^ The high bio‐oil yield is due to the thermochemical treatment of the organic waste at around 500 °C, with a fast‐heating rate from 300 to 800 °C min^−1^ and short residence time (≤10 s). Fast pyrolysis may not be suitable for all types of organic waste, as the particle size of the waste typically needs to be less than 3 mm for a high heat transfer rate at the particle interface, due to the low thermal conductivity of organic waste.^[^ ^48^ ^]^

**Flash Pyrolysis** is an improved version of fast pyrolysis with large‐scale applicability in sewage sludge valorization, generating bio‐oil as the primary output.^[^ ^49^ ^]^ It is conducted at a high temperature of 700–1100 °C, using an extremely high heating rate (≥1000 °C min^−1^) and very short residence time (≤2 s).^[^ ^47^, ^50^ ^]^

The main challenge for commercializing flash pyrolysis is configuring a reactor for organic waste that meets a high heating rate and short residence time requirements.^[^ ^51^ ^]^

**Vacuum Pyrolysis** is the thermal degradation of organic waste under low pressure (≤0.02 MPa) without oxygen, at ≈300–600 °C.^[^ ^47^, ^52^ ^]^ It is comparable to slow pyrolysis in terms of heating rate and residence time, suitable for organic vapour rapid removal and high bio‐oil yield. Vacuum treatment is advantageous in producing high‐porosity biochar.^[^ ^53^ ^]^ This process can pyrolyze larger particles without carrier gas.^[^ ^47^ ^]^ However, it is complex and expensive, requiring large vessels and piping to achieve high vacuum levels.

**Microwave‐Assisted Pyrolysis (MWP)** is a promising energy‐efficient, technique with minimal formation of hazardous products and emissions, for the conversion of organic waste into useful products, under an inert atmosphere.^[^ ^54^ ^]^ MWP offers a viable alternative to traditional pyrolysis methods due to benefits such as uniform heat transfer, rapid heating rate (5 to over 1000 °C min^−1^), instant on/off control, volumetric and consistent heating, and improved energy efficiency.^[^ ^54^, ^55^ ^]^ Extensive investigations have verified that MWP is better for producing biochar with high and uniform porosity.^[^ ^56^ ^]^ Lam et al. demonstrated the potential of MWP in converting orange peel into porous carbon with a total pore volume of 0.6 cm^3^ g^−1^ and surface area of 1350 m^2^ g^−1^.^[^ ^55^ ^]^ MWP is generally limited to particle sizes of 1–2 cm, presenting scaling‐up challenges.^[^ ^47^ ^]^ Additionally, microwave heating is more costly than traditional heating methods due to electricity costs.^[^ ^57^ ^]^

**Figure 4:**
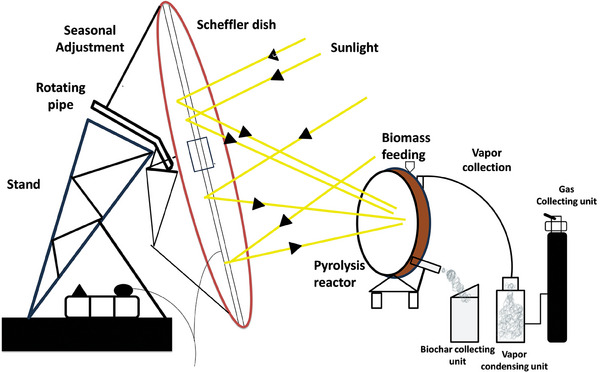
Solar‐assisted pyrolysis system used for converting non‐edible seeds to biochar. Adapted with permission.^[^ ^59^ ^]^ 2017,Elsevier.

**Solar‐Assisted Pyrolysis** involves optical devices to concentrate solar energy on a tubular reactor or directly on the material^[^ ^58^, ^59^ ^]^ (**Figure** **4**). This can be achieved by focusing the solar radiation on a small area through the walls of the reactor or direct feedstock irradiation.^[^ ^60^ ^]^ Solar‐assisted pyrolysis converts organic waste such as biomass into biogas, bio‐oil, and biochar.^[^ ^61^ ^]^ Solar concentrators of various capacities are used to redirect solar radiation energy from a large area and concentrate it on a smaller area producing a temperature as high as 1000 °C. Researchers have used different arrangements of the thermo‐solar reactor systems made up of collector, concentrator, reactor and support architectures to achieve different levels of efficiency.^[^ ^62^ ^]^ Solar‐assisted pyrolysis enables the exploitation of solar energy to provide the required energy to break the bonds of organic materials and form chemical commodities (such as bio‐fuel or tar and biochar).^[^ ^63^ ^]^ The utilization of high‐powered concentrators (dish receivers) helps to attain the initial pyrolysis temperature at a shorter time (high heat flux) compared to fossil fuel heating, and the rate of heating can be controlled.^[^ ^64^ ^]^ The rapid heating during solar‐assisted pyrolysis reduces the amount of time that tar vapour remains in pores and shortens the condensation reaction, preserving more functional oxygen and hydrogen in the biochar due to the low carbon content.^[^ ^65^ ^]^ Solar biomass pyrolysis produces gases with higher heating values per unit of feedstock, and they are cleaner compared to those generated by other heating methods.^[^ ^63^, ^66^ ^]^ As solar‐assisted pyrolysis can be done on‐site, this technology helps to overcome the biomass transportation challenges with no GHG emissions.^[^ ^66^, ^67^ ^]^ One of the challenges of solar‐assisted pyrolysis is biomass pre‐processing, which adds an initial cost to the process, along with the low density of solar energy that necessitates the use of concentrators or supplementary heat support for pyrolysis.^[^ ^68^ ^]^ The low optical absorption of biomass, due to its high reflectivity, is advantageous for producing more biochars than bio‐oil.^[^ ^69^ ^]^

#### 2.2.3 Torrefaction

Torrefaction is a mild pyrolysis process performed at 200–300 °C for 30–60 min in an inert atmosphere.^[^ ^70^ ^]^ It includes dry torrefaction (DT) and wet torrefaction (WT), also known as hydrothermal carbonization (HTC).

##### Dry Torrefaction (DT)

DT is effective for processing organic waste with low moisture content, while pre‐drying is necessary for waste with high moisture. DT is a promising pre‐processing step for improving the physicochemical properties of organic waste. Researchers have delved into the mechanism of DT, exploring reactions such as decarboxylation, dehydration, decarbonylation, demethoxylation, intermolecular rearrangements, condensation, and aromatization.^[^ ^70a^ ^]^ Torrefied organic waste from DT cannot be classified as biochar, as DT only marks the initial pyrolysis stages. Torrefied organic waste contains volatile matter and exhibits physicochemical properties between raw organic waste and biochar.

##### Hydrothermal Carbonization (HTC)

HTC is suitable for producing hydrochar from organic waste with high moisture content, typically over 50% (wet basis), due to pre‐mixing with water.^[^ ^71^ ^]^ HTC occurs in hot‐compressed water or subcritical water at 180 to 265 °C for 5 to 240 min, under a pressure of up to 6.0 MPa.^[^ ^72^ ^]^ A key product of this process is hydrochar, a carbon‐rich solid material. The yield of hydrochar is influenced by various factors: increasing the residence time of the feedstock in the reactor generally decreases the hydrochar yield, while a lower heating rate typically leads to higher hydrochar production.^[^ ^71^ ^]^

In the HTC processing of organic waste, hemicellulose is depolymerized into monomers and oligomers through hydrolysis, producing solid hydrochar with reduced moisture content.^[^ ^47c^ ^]^ Biochar is directly obtained through a dry carbonization treatment (i.e., gasification, pyrolysis), while hydrochar needs to be separated from the solution used for the hydrothermal carbonization process.^[^ ^39b^ ^]^ The physical and chemical characteristics of biochar and hydrochar significantly differ.^[^ ^39b^ ^]^

The hydrochar obtained from HTC is biologically sterilized and has a moderate heating value, high aromaticity, and porous structure.^[^ ^73^ ^]^ There is a growing environmental concern regarding the liquid output, which contains alkali compounds from the presence of varying nutrients that require appropriate treatment before disposal. Anaerobic digestion effectively reduces organic waste from HTC and produces methane‐rich biogas for energy recovery.^[^ ^74^ ^]^

#### 2.2.4 IonoThermal Carbonization (ITC)

Ionic liquids (ILs) are relevant for producing high‐porosity materials due to their low volatility, good solubility for organic waste, and thermal stability.^[^ ^75^ ^]^ The carbonization process utilizing ILs as catalysts and/or pyrogenic agents, is commonly known as ionothermal carbonization (ITC). ITC is easily conducted in an unsealed vessel at temperatures of ca 160–240 °C.^[^ ^75^ ^]^

Porous materials from ITC exhibit higher surface area with slightly greater carbonization yields compared to hydrochar.^[^ ^75c^ ^]^ Different Bmim (1‐Butyl‐3‐methylimidazolium)‐ type ILs with different anions (such as Cl^−^, BF_4_^−^, and Tf_2_N^−^) can be used to prepare value‐added materials.^[^ ^75d^ ^]^ The use of iron‐containing ILs (e.g., [Bmim][FeCl_4_]) as ionothermal solvents is widely reported for the preparation of porous value‐added materials.^[^ ^76^ ^]^ Despite its potential, ITC remains an emerging area with yet‐to‐be‐elucidated reaction mechanisms and an endless variety of carbon precursor/IL systems to explore.^[^ ^77^ ^]^ ITC‐derived materials, followed by nitrogen‐doping treatment such as post‐treatments with reactants like NH_3_ and HCN, are crucial for the development of carbon‐based functional materials.^[^ ^78^ ^]^ The nitrogen content can reach 8 wt% when [Bmim][BF4] and glucose are used as a solvent (nitrogen source) and precursor (carbon source), respectively.^[^ ^79^ ^]^ Although ILs can be recycled after ITC without additional purification treatment, their high cost could limit their widespread industrial applications.

**Table** **1** provides a comprehensive comparison of various thermo‐chemical methods employed for the valorization of organic waste. It details the characteristics of the carbon produced through each technique, emphasizing the distinct properties and performance of the resultant materials. This thorough examination facilitates the understanding of the advantages and limitations inherent in each method, aiding in the assessment of their effectiveness in converting organic waste from MSW into valuable carbon products.

**Table 1:** Comprehensive comparison of the thermo‐chemical methods for organic waste valorization.

Method	Process parameters	Organic waste characteristics	Output characteristics	Biochar characteristics	Advantages	Disadvantages
Gasification	Temperature: 800–1200 °C, ER < 1; Gasifying agents: air, oxygen, steam; High temperatures in oxygen‐deficient conditions	Suitable for carbonaceous materials (e.g., biomass, coal); Uniform feedstock required	85% syngas (H₂, CO), 5% tar/oil, 10% wt% biochar	Low porosity; low biochar yield; suitable for energy storage and catalysis due to physicochemical properties	Produces high‐quality syngas; versatile applications	Requires high operating temperatures; tar formation; complex technology
Slow Pyrolysis	Temperature: 350–900 °C; Heating rate: 5–7 °C/min; Residence time: long (≤1 h); Inert atmosphere	Suitable for various organic waste, including high‐carbon content	High biochar yield (>12 wt%); bio‐oil and syngas as byproducts	High stability and surface area; used in soil remediation, sorbents, and fertilizers	Produces high biochar yield; energy‐efficient	Long residence times; lower yield of bio‐oil/gas
Fast Pyrolysis	Temperature: ≈500 °C; Heating rate: 300–800 °C/min; Residence time: ≤10 s	Requires small particle sizes (≤3 mm) for efficient heat transfer	Up to 75 wt% bio‐oil, ≈12 wt% biochar, remaining as syngas	Lower biochar yield; moderate porosity; energy storage potential	High bio‐oil yield; suitable for biofuel production	Not suitable for large‐sized feedstock; high energy input
Flash Pyrolysis	Temperature: 700–1100 °C; Heating rate: ≥1000 °C/min; Residence time: ≤2 s	Applicable to sewage sludge and other fine‐grain organic waste	Bio‐oil as primary product; small yield of syngas and biochar	Limited application due to extremely short processing time	Large‐scale applicability; efficient bio‐oil production	Reactor design challenges; scalability issues
Vacuum Pyrolysis	Temperature: 300–600 °C; Pressure: ≤0.02 MPa; Slow heating and long residence time	Capable of processing larger particles without carrier gas	High yield of bio‐oil; biochar with high porosity	High porosity and surface area; suitable for high‐value applications	Suitable for larger feedstock; high bio‐oil quality	High operational complexity and cost
Microwave Pyrolysis	Rapid heating rate: 5–1000+ °C/min; Uniform heating; Inert atmosphere	Suitable for smaller particles (1–2 cm); requires uniform distribution of microwaves	High‐quality biochar with uniform porosity; bio‐oil and syngas as byproducts	High and uniform porosity; suitable for energy and catalytic applications	Energy‐efficient; minimal emissions; high product uniformity	High electricity cost; scaling up challenges
Solar Pyrolysis	Temperature: up to 1000 °C; Solar concentrators required; Renewable energy source	Biomass and other renewable feedstock with low reflectivity	High‐quality biogas; bio‐oil and biochar	Lower carbon content but retains functional groups; suitable for cleaner energy production	On‐site processing; zero emissions; renewable energy utilization	Biomass preprocessing costs; low energy density; dependency on sunlight
Dry Torrefaction	Temperature: 200–300 °C; Residence time: 30–60 min; Inert atmosphere	Suitable for low‐moisture organic waste; Pre‐drying required for high‐moisture content	Preprocessed organic waste; intermediate volatile matter	Properties between raw organic waste and biochar; not classified as biochar	Improves feedstock characteristics for further processing	Requires additional drying step for moist feedstock
Hydrothermal Carbonization (HTC)	Temperature: 180–265 °C; Pressure: ≤6.0 MPa; Residence time: 5–240 min; Water‐based processing	Ideal for wet feedstock with >50% moisture content	Hydrochar with high aromaticity and porous structure; moderate heating value	Moderate porosity; sterilized; used in energy and soil remediation	Suitable for high‐moisture waste; produces hydrochar with a moderate heating value	Liquid effluent requires treatment; lower yield compared to pyrolysis
**Ionothermal Carbonization (ITC)**	Temperature: ≈160–240 °C; Uses ionic liquids as catalysts/solvents	Requires well‐suited carbon precursors and ionic liquids	High‐porosity carbon materials; nitrogen‐doped products possible	High porosity and surface area; customizable for advanced applications	Recyclable ionic liquids; nitrogen doping capability	High cost of ionic liquids; limited industrial scalability

Biochar and carbon materials derived from organic waste valorization have emerged as versatile resources for energy storage and catalysis applications, owing to their high surface area, tunable porosity, functionality, and robust chemical stability. In energy storage, these carbon materials are commonly used as electrodes in supercapacitors and batteries. Their structural properties, including high electrical conductivity, customizable pore distribution, and functionality, enhance charge storage capabilities and improve energy and power densities, making them ideal for advanced energy systems.^[^ ^80^ ^]^

In catalysis, biochar and derived carbon materials serve as effective supports for metal nanoparticles or as standalone catalysts. They are employed in various reactions, including the oxygen reduction reaction (ORR), hydrogen evolution reaction (HER), methane (CH_4_), and carbon dioxide (CO₂) conversion processes. Their surface functionalities, such as oxygen‐ and nitrogen‐containing groups, can participate in the catalytic activity and selectivity.^[^ ^81^ ^]^

## 3 Advanced Valorization of Plastic Waste and Metal Waste into MOFs

**Scheme 1:**
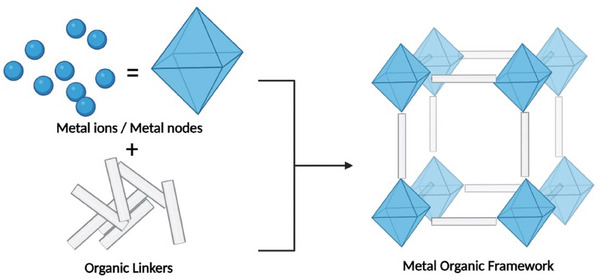
Assembly of metal nodes and organic linkers to form an MOF.

MOFs, a hybrid class of porous crystalline materials, are formed by connecting metal nodes, comprising metal ions or metal cluster units, with organic linkers (**Scheme** **1**). MOFs possess characteristics of both their organic and inorganic components.^[^ ^82^ ^]^

These materials have caught the attention of researchers because of their large pore sizes and surface areas, and ability to withstand heat and pressure.^[^ ^83^ ^]^ Their applications include CO_2_ capture and conversion,^[^ ^84^ ^]^ water treatment,^[^ ^85^ ^]^ sensing,^[^ ^86^ ^]^ and gas separation.^[^ ^87^ ^]^ The primary challenge in synthesizing these materials lies in the small‐scale (gram scale) production and the high cost of the starting materials. To address these limitations, recent research has focused on utilizing waste materials, such as PET from plastic, and waste metals (including those from e‐waste), as sustainable precursors for MOF synthesis.

**Scheme 2:**
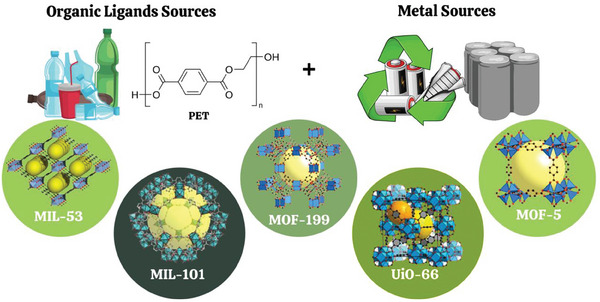
Schematic of PET and metal waste‐derived MOFs.

Deriving MOFs from plastic waste, particularly PET, involves innovative processes. PET is depolymerized to extract its organic monomer, BDC. This monomer serves as a linker, combining with metal ions to form the MOF structures. BDC is frequently used as linker in the synthesis of MOFs^[^ ^88^ ^]^ such as MOF‐5,^[^ ^89^ ^]^ UiO‐66,^[^ ^90^ ^]^ MIL‐53,^[^ ^91^ ^]^ and MIL‐101^[^ ^92^ ^]^ (**Scheme** **2**).

The process can either separate hydrolysis and synthesis into distinct stages or integrate them into a single, continuous step. Innovative solvent‐free methods like mechanochemical milling offer scalable, eco‐friendly alternatives. Emerging techniques like mechanochemical milling eliminate the need for solvents, enhancing scalability and reducing environmental impact. The resulting MOFs find applications in catalysis, pollutant removal, energy storage, and beyond, demonstrating a sustainable pathway for plastic waste valorization​.

Previous studies have confirmed the feasibility of transforming PET and/or metal waste into MOFs.^[^ ^93^ ^]^ Studying green, efficient, and scalable methods for this conversion is crucial for advancing sustainable chemical processes for waste valorization.

### 3.1 Organic Linkers Derived from PET Waste

Recovered chemicals can be employed to produce valuable materials. For instance, BDC, derived from the depolymerization of PET, is extensively utilized in the synthesis of MOFs.^[^ ^14^, ^15^ ^]^ The synthesis of MOFs from waste PET can be achieved through either a two‐step process or a one‐pot synthesis.

The two‐step approach involves the hydrolysis of PET into BDC, followed by the purification of the obtained organic linker which is then combined with metal salts to construct MOFs. One‐pot synthesis process involves hydrolyzing PET and directly synthesizing MOFs in a single continuous procedure.^[^ ^94^ ^]^ Since the process of PET hydrolysis is often accelerated by employing acid catalysts or bases, like sulfuric acid or potassium hydroxide, there is the possibility that the pH of the solution changes, thus disrupting the MOF synthesis process. Conducting hydrolysis separately allows for pH adjustment and purification of the BDC before its utilization in MOF synthesis. Nevertheless, the additional steps involved in the process and the generation of salt waste can outweigh the benefit of using PET as a source of linkers.

An early study showed the successful synthesis of a series of robust MOF structures (e.g., Al‐MIL‐53, V‐MIL‐47, Cr‐MIL‐53, Fe‐MIL‐88B, and Zr‐UiO‐66), using waste bottle plastic as the source of organic linkers obtained through PET hydrolysis.^[^ ^95^ ^]^ When PET hydrolysis is conducted in a one‐pot microwave‐assisted process under hydrothermal conditions to produce BDC without purification and without Dimethylformamide (DMF) solvent under hydrothermal conditions, both aluminium‐based MIL‐53 (Al‐MIL‐53) and vanadium‐based MIL‐47 (V‐MIL‐47) are simultaneously synthesized. However, low‐stability MOF structures like Fe‐MIL‐88B, are only obtained when the reaction is carried out in two separate steps, involving linker extraction and isolation. Other studies showed the successful synthesis of pure and highly defective UiO‐66 and its functionalized version UiO‐66‐NO_2_ via one‐pot PET‐to‐MOF upcycling process under mild conditions.^[^ ^96^ ^]^ Very recently, three types of MOFs, Fe‐, La‐, and Zr‐based MOFs, were constructed using recycled PET bottles for eco‐friendly and cost‐effective arsenate removal. These MOFs were synthesized using a solvothermal approach, without the need for purification and separation of the linker.^[^ ^97^ ^]^

One‐pot and two‐step synthesis techniques were mainly conducted under solvothermal, hydrothermal, and microwave conditions. These conventional solvothermal techniques face challenges such as excessive use of toxic solvents, prolonged reaction durations, elevated pressure, and high temperatures, hindering the large‐scale production of MOFs from waste PET. Therefore, there is an urgent requirement for innovative, environmentally friendly, and scalable approaches to convert PET into a variety of MOFs for sustainable chemical processes. Achieving this remains a significant challenge.

Mechanochemical milling, as described by He et al., represents a transformative approach for converting PET waste into valuable MOFs.^[^ ^98^ ^]^ This method uses mechanical energy to induce chemical reactions without the need for solvents, thereby bypassing the aforementioned environmental and safety issues associated with traditional solvothermal synthesis.^[^ ^99^ ^]^ In the mechanochemical process described, PET waste is first mechanically milled in the presence of sodium hydroxide to break down its polymer chains into smaller BDC segments. These segments then act as organic linkers that coordinate with various metal ions, also introduced during the milling process, to form MOFs with yields of 54–90%, at room temperature, in a relatively short time. For example, Tang et al. introduced an innovative method for transforming waste PET into various MOFs using mechanochemical milling.^[^ ^98^ ^]^ Waste PET served as a cost‐effective source of BDC for the MOF synthesis. The resulting MOFs are agglomerated nanoparticles with a distinct crystal structure. The inferred growth mechanism suggests that BDC coordinates with metal ions to form nanosized fragments, which then assemble into the MOF framework and bulk structures.

**Scheme 3:**
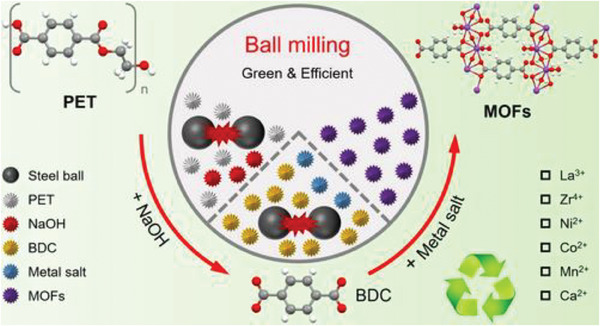
Eco‐friendly transformation of PET into various metal–organic frameworks (MOFs) through ball milling at ambient temperature. Adapted with permission.^[^ ^98^ ^]^ 2023,Wiley‐VCH.

The environmentally friendly and efficient mechanochemical milling technique enables large‐scale MOF production. For instance, they successfully produced ≈60.1 g of Ni‐MOF in the laboratory, demonstrating the significant potential for commercializing MOFs derived from waste PET.^[^ ^98^ ^]^ PET powder was initially depolymerized into BDC via solid‐state alkaline hydrolysis. Following this, BDC was coordinated with various metal ions (e.g., La^3+^, Zr^4+^, Ni^2+^, Co^2+^, Mn^2+^, and Ca^2+^) yielding La‐MOF, Zr‐MOF, Ni‐MOF, Co‐MOF, Mn‐MOF, and Ca‐MOF (**Scheme** **3**).^[^ ^98^ ^]^ Similarly, Guo et al. proposed an eco‐friendly synthesis method to create defective UiO‐66 (d‐UiO‐66). They combined ZrOCl_2_·8H_2_O, PET‐derived BDC, and benzoic acid in an agate mortar and ground the mixture for 15 min at room temperature. After heating the resulting solid, the mixture was soaked in ethanol at 343 K, for 24 h. The solids were collected, sequentially washed with DMF and ethanol, and then dried at 353 K to produce d‐UiO‐66, which is effective in removing lomefloxacin (LOM) from water. This solvent‐free process yields d‐UiO‐66 with higher porosity and more defective Zr sites compared to defect‐free UiO‐66, significantly enhancing LOM adsorption.^[^ ^100^ ^]^ The technique's eco‐friendliness and cost‐effectiveness stem from its solvent‐free nature and ability to operate under ambient conditions, which not only reduces energy consumption but also simplifies the scaling up of production for industrial applications. Moreover, mechanochemical milling enhances the feasibility of continuous processing, which is pivotal for large‐scale manufacturing.

### 3.2 Metal Wastes and E‐Waste as Precursors for MOF Synthesis

#### 3.2.1 Metal Wastes

As previously mentioned, developing sustainable processes for recovering and synthesizing valuable products from metal wastes is critical for both economic and environmental reasons. For instance, researchers have focused on recycling and upcycling various metal‐based wastes, such as mining residues, oil refinery byproducts, e‐waste, and other forms of metallic wastes. Using these metal‐based wastes as raw materials offers a sustainable method for creating value‐added materials.

Acquiring eco‐friendly methods to recycle and upcycle valuable substances from solid waste is crucial for both economic and environmental well‐being. In this context, the scientific community is increasingly focused on transforming metal‐rich waste from industries such as mining, electronics, and oil refining into useful raw materials. These materials are then used to create MOFs with enhanced value, exemplifying the potential for everyday waste to be transformed into beneficial new products. Building on this, recent studies have extended their focus to include not only the use of cost‐effective alternative precursors but also inducing environmental protection. This has led to the synthesis of various MOFs such as MIL‐101(Cr), MIL‐53(Cr), and MOF‐5, all derived from solid waste.^[^ ^101^, ^102^ ^]^ For instance, Lu et al. reported a synthesis of MIL‐101(Cr) from metal waste utilized waste chromium as the source, employing Cr^6+^found in industrial wastewater.^[^ ^101^ ^]^ The process started with the chemical reduction of Cr^6+^ to Cr^3+^ using sulfite at room temperature. This was followed by the environmentally friendly synthesis of MIL‐101(Cr), which involved mixing the Cr^3+^solution with BDC under solvothermal conditions in a sealed vessel, without the use of hydrofluoric acid (HF), typically used in traditional methods. This method not only recycles waste chromium, reducing environmental consequences, but also produces MIL‐101(Cr) with properties comparable to those prepared from pure Cr^3+^ sources, including high surface area and pore volume suitable for applications like carbon monoxide separation.

Another study presented a method to synthesize aluminium‐based MOFs using aluminium sources, including aluminium foil and waste Coca‐Cola cans.^[^ ^103^ ^]^ Initially, they used highly pure aluminium foil with terephthalic acid in a one‐pot solvothermal process to produce nanocrystals of MIL‐53(Al) on the foil surface. The aluminium foil acted both as a support medium for the growth of the MOF/aluminium composite and as an insoluble precursor for creating porous materials. Expanding this approach, the team then used recycled aluminium from waste Coca‐Cola cans as a source to synthesize MIL‐53(Al) and MIL‐96(Al) using terephthalic and trimesic acids, respectively. Despite the presence of numerous impurities in the recycled cans, the synthesis was successful, and the resulting MOFs were highly microporous.

#### 3.2.2 E‐Waste

Waste Printed Circuit Boards (WPCBs) are a crucial part of e‐waste and have a diverse composition.^[^ ^104^ ^]^ WPCBs account for about 10% of the total e‐waste generated. WPCBs are particularly valuable due to their high concentrations of metals like copper (Cu) and tin (Sn); precious metals such as silver (Ag), gold (Au), and palladium (Pd)^[^ ^105^ ^]^; and rare earth elements.^[^ ^106^ ^]^ However, it is crucial to note that e‐waste also contains hazardous elements such as cadmium (Cd), mercury (Hg), nickel (Ni), and lead (Pb).^[^ ^107^ ^]^ Improper handling and disposal of these toxic substances pose severe health and environmental risks, underscoring the need for effective and safe recycling practices.

The recovery of metals from WPCBs is an appealing applied research area due to the high concentration of metals in this type of waste. From an economic perspective, transforming metal‐containing waste into MOFs is highly advantageous. In this context, Gholami et al. demonstrated an innovative approach to e‐waste recycling by combining leaching solutions (H_2_SO_4_, CuSO_4_, and NaCl) with an electrochemical method to extract copper from waste printed circuit boards.^[^ ^108^ ^]^ The recovered copper was used to synthesize a magnetic metal–organic framework (mag‐MOF) based on 2‐methylimidazole (2‐MIM) linker, which was then incorporated into a TiO_2_/mag‐MOF(Cu) composite. This composite was tested for its photocatalytic activity in degrading organophosphorus pesticides malathion (MTN) and diazinon (DZN). The catalyst showed excellent recovery and stability over five cycles, achieving over 83% and 85% mineralization for MTN and DZN, respectively.^[^ ^108^ ^]^ It also proved effective in treating tap water and wastewater samples, highlighting its potential as a catalyst for photodegradation in aqueous solutions.

In Section 5, we will focus on green hydrometallurgy approaches to metals recycling from e‐waste.

### 3.3 Direct Synthesis of MOFs from Combined Waste Streams

To achieve a circular economy and practical applicability of the “waste‐to‐MOF” approach, a complete synthetic approach of the MOF crystals starting with waste sources for both the organic linker and metal centers is required. In this case, a sustainable, cost‐effective, and scalable MOF synthesis must be achievable. By using PET waste plastic bottles as an organic linker source and lithium‐ion‐battery (LiB) waste as a source of metal ions, Al‐MOF (MIL‐53) was successfully produced. It is noteworthy to mention that LiB waste generates Al metal ions in addition to other metals such as Mn, Cu, Ni, Li, and Co. Nevertheless, only MIL‐53 was selectively precipitated by the combined solutions of terephthalic acid and recovered metals under solvothermal techniques.^[^ ^109^ ^]^ This MOF was also synthesized by employing aluminium foil or beverage cans as an Al source and PET water/beverage bottles as the linker's source.^[^ ^110^ ^]^ The so obtained MOF was shown to be porous and the CO_2_ adsorption and N_2_ uptake were similar to those of the conventional one.

A very recent study showed that Al‐MIL‐53 can be directly synthesized from photovoltaic (PV) waste. Indeed, PET in the polymeric back sheet and Al from the solar cells were used as linker and metal source, respectively, to generate MIL‐53(Al).^[^ ^111^ ^]^ WPCB wastewater and alkali reduction wastewater (AR) were simultaneously employed as Cu and BDC sources, to synthesize Cu‐based MOF^[^ ^112^ ^]^ in a quantitative yield of 75.5%. Recently, Deka et al. have synthesized a Fe‐based MOF material using rust as the metal source and PET bottles as the BDC linker source via a solvothermal technique. Although acid and base treatments were needed for the precursor extraction and purification, this approach does not typically put a significant load on the environment when proper measures are taken into consideration, such as working under mild conditions.^[^ ^113^ ^]^ Finally, green and sustainable approaches were employed to synthesize a variety of Ca‐based MOFs by using water‐based and mechanochemical synthesis techniques from eggshells as a source of Ca(II) ions and recycled PET bottles as precursors for the BDC ligands.^[^ ^114^ ^]^

Transforming electroplating sludge (EPS) and PET waste into MOFs is advantageous for both reducing pollution and promoting a sustainable economy. Lin et al presented a novel green synthesis of Ni‐MOF nanocrystals by using EPS and PET waste as precursors. Remarkably, even with the full utilization of EPS, containing abundant Fe^3+^/Cu^2+^ ions alongside Ni^2+^ ions, the fabrication of Ni‐MOFs was successful.^[^ ^115^ ^]^ These Ni‐MOF nanocatalysts demonstrated outstanding activity in the photoreduction of CO_2_, achieving a CO production rate of 9.68 × 10^3^ µmol h^−1^ g^−1^ with a 96.7% selectivity over H_2_ evolution.^[^ ^115^ ^]^ Additionally, the apparent quantum yield at 420 nm was 1.36%, outperforming most catalysts in similar systems. This work shows that high‐performance MOF nanocrystals with excellent photocatalytic properties can be effectively produced from solid wastes.

Coupling organic and inorganic wastes to produce MOFs appears to be the promising, eco‐friendly, and cost‐effective technique to upcycle plastic and metal wastes expanding the possibilities for various combinations of byproducts to be utilized with the goal of producing functional materials and scaling them up. However, challenges arise concerning the purity of the obtained precursors and extraction methods required to isolate usable chemicals from plastic waste and metal ions from metal waste, which are crucial for the quality and functionality of the resulting MOFs.

### 3.4 Cost‐Effectiveness and Feasibility of Transforming Waste into MOFs

The most significant barriers to MOF commercialization include the necessity for high energy, the challenge of achieving stable products, and the limitation of low output.^[^ ^116^ ^]^ Roughly 70 000 metal–organic frameworks are documented,^[^ ^117^ ^]^ with standard synthesis procedures producing less than one gram per batch to ensure high crystal quality. Indeed, cost considerations stand as one of the main concerns in the commercialization process of MOFs. In the case of MOF‐5, for instance, one kilogram of activated MOF‐5, requires 81.30 L of DMF, 1.03 kg of terephthalic acid, and 3.45 kg of zinc acetate dihydrate, with an overall yield of 63%.^[^ ^118^ ^]^ The direct material cost for MOF‐5 scale‐up is 527.3 USD per kilogram, with the solvent representing 78.6% of the total cost.^[^ ^116^ ^]^ To make MOF‐5 financially viable in the market, strategies like recycling solvents, utilization of less expensive solvents, and the use of more concentrated solutions are essential to lower expenses. Building on this, Boukhvalov et al. emphasized that manufacturing 1 kg of W‐MOF‐5 could be economically efficient, with costs dropping to 42 USD when accounting for more than 90% recovery of the solvent used.^[^ ^119^ ^]^ Expanding on the economic implications discussed, it's noteworthy that producing 1 kg of MIL‐53(Al) from waste PET bottles and aluminium foil costs only a third of the price charged by Sigma‐Aldrich for the same MOF.^[^ ^110^ ^]^ Further cost analysis reveals that producing 1 kg of non‐supported MIL‐53(Al) using metallic aluminium amounts to only $3.49. This compares dramatically to the $10 000 per kg price for the same material sold by BASF, highlighting the significant cost benefits of using recycled materials.^[^ ^103^ ^]^ Economically, MOFs offer substantial opportunities in urban mining by improving the extraction of valuable materials from e‐waste streams. This is particularly beneficial in regions where urban mining can stimulate economic growth and reduce dependency on natural resource extraction. MOFs enhance the recovery of precious and rare earth metals, which are abundantly present in discarded electronics through direct synthesis or selective adsorption.^[^ ^120^ ^]^

### 3.5 Precious Metals Recovery by Adsorption Using MOFs

One of the key advantages of MOFs in e‐waste recycling is their ability to selectively adsorb precious metals from complex waste streams.^[^ ^121^ ^]^ MOFs can be designed with specific functional groups, such as thiols or amines, to target these valuable metals. For instance, MIL‐101(Cr) has shown great potential in aqueous‐phase adsorption for recovering critical elements like rare earth metals and uranium.^[^ ^122^ ^]^ This MOF was modified through two distinct methods using a carbamoylmethylphosphine oxide‐type ligand (CMPO): one method involved anchoring the ligand covalently to the MOF surface in a three‐step process, while the other one encapsulated the ligand within the MOF cages to prevent leaching.^[^ ^122^ ^]^ Another example is by Ding and coworkers who presented novel materials, termed FGFAM/FGFTM hybrids, which combine 3D functional graphene foam with amino‐/thiol‐based copper MOFs (Cu‐pPDA/Cu‐pPDT).^[^ ^123^ ^]^ These hybrids are designed for selectively recovering Au^3+^ and Pd^2+^ from solutions. They demonstrate exceptional adsorption capacities, achieving 3714.8 mg g^−1^ for Au^3+^ and 3344.6 mg g^−1^ for Pd^2+^. The superior performance is attributed to the diverse functionalities within the hybrid frameworks, fostering interactions such as hydrogen bonding, electrostatic interactions, and inner‐sphere complexation with Au^3+^ and Pd^2+^. Moreover, the 3D graphene foam provides a large surface area and unique morphology that supports MOF growth, particularly enhancing the active site availability of Cu‐pPDT MOF. The study also demonstrates effective recovery of Au^3+^ and Pd^2+^ from different solutions, achieving 98–99% recovery even after five cycles using thiourea/HCl solution.^[^ ^123^ ^]^

### 3.6 Sequestration of Rare Earth Elements (REEs) by MOFs

Rare earth elements (REEs) such as neodymium, dysprosium, and terbium are critical for high‐tech and energy applications. MOFs can be tailored to selectively recover these elements from e‐waste leachate. The ability to design MOFs with specific pore sizes and functional groups allows for the targeted adsorption of REEs, facilitating their separation and recovery. This approach not only conserves these critical materials but also reduces the environmental impact associated with traditional mining practices. For example, Vigneswaran's group synthesized a chromium‐based metal–organic framework (Cr‐MIL‐PMIDA) modified with N‐(phosphonomethyl)iminodiacetic acid (PMIDA) for the selective recovery of europium (Eu) from chemically complex zinc ore leachate.^[^ ^120c^ ^]^ The Cr‐MIL‐PMIDA exhibited a maximum adsorption capacity of 69.14 mg g^−1^. Importantly, the material demonstrated high selectivity (88%) for Eu over competing transition metal ions commonly found in the leachate.^[^ ^120c^ ^]^ The study also highlighted the structural stability of Cr‐MIL‐PMIDA over multiple regeneration cycles, suggesting its potential for industrial applications in REE recovery. Wang et al. demonstrated the application of HKUST‐1 for recovering rare earth ions (Ce^3+^ and La^3+^) from aqueous solutions, underscoring their importance as valuable resources.^[^ ^124^ ^]^ HKUST‐1 exhibited robust affinity and selectivity, achieving notable capacities of 234 mg g^−1^ for Ce^3+^ and 203 mg g^−1^ for La^3+^ at pH 6. The adsorbent displayed ≈87% selectivity for rare earth ions over other competing metal ions.^[^ ^124^ ^]^

## 4 From Hydrometallurgy to Green Hydrometallurgy

### 4.1 Hydrometallurgical Process

**Metallurgy** has been practiced for millennia and has marked significant evolutionary steps for humanity. In recent years, e‐waste recycling technologies have advanced considerably due to the tireless efforts of researchers committed to sustainability. Currently, the most common methods for recovering metals from e‐waste are pyrometallurgy and hydrometallurgy. Biometallurgy, which leverages the metabolic pathways of microorganisms, has lower technological readiness due to the microbes' excessive sensitivity, limiting the processing capacity of WEEE in a particular batch.^[^ ^125^ ^]^ Most pyrometallurgical reactions are endothermic, requiring heat supplied by combustion or, less frequently, by electricity (induction, radiation, conduction, or plasma). The process produces metal alloys, oils from plastics, and large amounts of solid waste. Incineration during pyrometallurgy of organic compounds generates significant CO_2_ and other volatile and toxic pollutants such as dioxins, furans,^[^ ^126^ ^]^ and dust. Lead vapours, which cause neurological, immunological, and hematological disorders,^[^ ^127^ ^]^ are also released. While emissions can be partially mitigated through step‐by‐step, selective, and fast heating processes,^[^ ^128^ ^]^ large‐scale processes remain very energy‐intensive, pose serious environmental problems, and lack selectivity.

**Hydrometallurgy** relies on aqueous solutions to recover metals. When organic and ionic liquid/salt eutectic solutions are used, the techniques are called solvometallurgy and ionometallurgy, respectively. A prerequisite for hydrometallurgical processing is crushing scraps to a particle size of less than ≈2 mm and physically separating the metal fraction for optimal extraction. The hydrometallurgical process involves three main phases: leaching, purification, and metal recovery.

The first step in the hydrometallurgical process is metal dissolution through oxidative leaching using suitable oxidants and, possibly, complexing agents.

**Table** **2** illustrates the conventional chemical strategies for the oxidative leaching of gold, the least oxidable metal in e‐waste.^[^ ^129^ ^]^ Leaching is a solid‐liquid extraction of metal ions, and the ease with which atoms enter the solution in the cationic state depends on the metal's inertness. Acid leaching with hydrogen evolution is effective for base metal oxidation, but protons cannot capture the external electrons of noble metal atoms. Therefore, most processes require a complexing agent in the reaction mixture. Further, the oxidative transfer of various metals in e‐waste into the same leachate results in a solution loaded with diverse cations and/or metal complexes. Since e‐waste contains both base metals (especially copper) and noble metals resistant to oxidation, harsh leaching chemistry is necessary to break down the most stubborn metal bonds of noble metals.

**Table 2:** Common chemical strategies for the oxidative leaching of gold.^[^ ^125^, ^176^, ^177^ ^]^

Oxidant	Complexing/ chelating species	Reaction	Disadvantage	pH
HNO_3_	Cl^−^	2Au+11HCl+3HNO_3_ = 2HAuCl_4_+3NOCl+6H_2_O (aqua regia)	Corrosiveness no scale‐up. The same can be said for the modified piranha solution (H_2_SO_4_/H_2_O_2_/HCl)	very acidic
O_3_	Cl^−^	2Au+3O_3_ + 6H^+^ + 8Cl^−^→2AuCl_4_^−^ +3O_2_+3H_2_O	Electricity consumption for O_3_ generation (4‐8 kWh kg^−1^ metal leached)	very acidic
O_2_	CN^−^	4Au+8CN^−^+O_2_+2H_2_O = 4Au(CN)_2_^−^+4OH^−^	Toxicity	>10
X_2_ (X = Cl, Br, I)^b^ or HClO_3_	X^−^	2Au+3Cl_2_+2Cl^−^ = 2AuCl_4_^−^	Cl_2_ toxicity and corrosiveness prevent scale‐up; Iodine is more user‐friendly but more expensive.	weak acidic
Fe^3+^	SCN^−^	Au +2SCN^−^ + Fe^3+^ = Au(SCN)_2_^−^ +Fe^2+^		very acidic
O_2_/Cu(NH3)_4_^2+^ ^a^	S_2_O_3_^2−^	4Au+8S_2_O_3_^2−^+O_2_+2H_2_O = 4[Au(S_2_O_3_)_2_]^3−^+4OH^−^	Low stability of thiosulfate	9‐10.5
Fe^3+^	CS(NH_2_)_2_	Au+2CS(NH_2_)_2_+Fe^3+^ = Au(CS(NH_2_)_2_)^+^+Fe^2+^	Low stability and carcinogenicity of thiourea	1‐2

a Cu(NH_3_)_4_^2+^ is used as a catalyst.

b Only Cl is used at the industrial scale.

Aqua regia exemplifies the conventional leaching solution composition, containing an oxidant (nitric acid) and a complexing species (chloride from hydrochloric acid). The complexing agent coordinates with the metal, keeping the resulting cation in solution and facilitating oxidative dissolution by the oxidant. However, aqua regia's corrosiveness poses significant risks to workers and the environment, making industrial‐scale reactors impractical and economically unfeasible. Similar concerns apply to the modified Piranha solution (H_2_SO_4_/H_2_O_2_/HCl).

The cyanide‐based MacArthur‐Forrest process, the industrial standard for over a century for leaching gold and silver from ores, has also been adopted in urban mining.^[^ ^130^ ^]^ Despite its toxicity, cyanide was once claimed to be a beneficial and environmentally friendly lixiviant at higher pH levels.^[^ ^131^ ^]^ Significant efforts have been made to find less toxic cyanide substitutes, such as thiosulfate, thiourea, disubstituted thiourea, thiocyanate, halides, and alternative oxidants like ozone, halogens, hypochlorite, or metals in high oxidation states. Aqueous iodine‐iodide leaching offers safety and environmental benefits, but the high cost of reagents hinders economic viability, even with the assistance of environmentally benign H_2_O_2_.^[^ ^132^ ^]^

Exotic and expensive sulfur‐donor ligands have been explored in various media, including organic solvents, ionic liquids, deep‐eutectic solvents (DESs), and water. However, the need for complex ligands prevents these methods from scale‐up and the use of organic solvents reduces the greenness of the method.

The stability constants of noble metal complexes with the indicated ligands suggest almost quantitative gold recoveries only in the most aggressive leaching environments, such as aqua regia, halide, and cyanide solutions.^[^ ^133^ ^]^ Thiourea and thiosulfate have moderate environmental impacts but are not economically viable, and their technologies are less reliable, preventing scale‐up. Halide leaching shows high recovery and moderate toxicity, but corrosion issues and technological reliability need to be addressed.^[^ ^134^ ^]^

The second step in hydrometallurgy involves purifying the leachate to enable the selective recovery of leached metal ions through ion exchange, solvent extraction, chemical precipitation, and/or biometallurgy. The harsh chemistry of the initial leaching phase makes this purification mandatory, yet it remains challenging due to limited selectivity.

Innovative approaches have been explored, such as host–guest supramolecular interactions with α‐cyclodextrins or cucurbiturils,^[^ ^135^ ^]^ porphyrin‐based polymers,^[^ ^136^ ^]^ macrocyclic tetralactam receptors,^[^ ^137^ ^]^ and niacin for the selective precipitation of noble metals from e‐waste aqua regia leachate.^[^ ^138^ ^]^ A tertiary diamide precipitated gold selectively from aqua regia leachates of e‐waste, achieving a 70% gold uptake with minimal removal of other metals in the presence of 29 metals in 2 M HCl.^[^ ^139^ ^]^ Similarly, gold was recovered quite selectively from a mixture of metals representative of e‐waste leachate by solvent extraction with an amide carrier.^[^ ^140^ ^]^

Some exotic adsorbents, such as 2‐mercaptobenzimidazole loaded onto chitosan microparticles, have been used to capture precious metals from the leachate.^[^ ^141^ ^]^ Various custom‐made ion exchange resins (chitosan‐based or glycidyl methacrylate/divinylbenzene‐based) featuring a wide range of active functionalities have also been reported.^[^ ^142^ ^]^ However, these lab‐scale strategies, requiring very specific chemicals, are difficult to industrialize and unreasonably aim to regain selectivity after the unselective leaching of precious and base metals.

The final step is the reductive transformation of the soluble metal ion into its elemental recoverable form. Cementation is useful but introduces immolative base metal ions into the solution.^[^ ^143^ ^]^ Guo et al. photoreduced gold selectively using carbon nitride (C_3_N_4_).^[^ ^144^ ^]^ Electrometallurgy employs electrolysis to produce metals by cathodic deposition from solutions,^[^ ^143^ ^]^ with renewable sources of electricity potentially improving the sustainability of this technology.

These three steps of the hydrometallurgical strategy are carried out at moderate or low temperatures, making it more energy‐efficient and sustainable compared to pyrometallurgy.^[^ ^145^ ^]^ Additionally, hydrometallurgy produces less toxic gas and dust, has easier lab execution, and lower operating costs.^[^ ^131^ ^]^ Thus, it is considered suitable also for small‐scale applications.^[^ ^134^ ^]^ However, the lower process temperatures result in slower kinetics, the use of synthetic reagents, and significant effluents are major disadvantages.^[^ ^131^ ^]^ The current hydrometallurgical strategy inherently decreases the purity of the recovered metals since the lack of systematicity in the first oxidative leaching step cannot be completely remedied by subsequent selective phases.

### 4.2 Attempts Toward Green Hydrometallurgy: Lower Toxicity and Selective Dissolution

To develop more sustainable leaching strategies, a diverse array of reagents and intricate sequential oxidative chemistries have been devised. In electrochemistry and solution chemistry, the stabilities of materials under standard conditions are described by Pourbaix diagrams. These diagrams are useful for assessing the stabilities of corrosion products, including derived oxides, hydroxides, oxyhydroxides, and free metal ions in solution.^[^ ^146^ ^]^ However, they do not provide kinetic information on actual corrosion rates. The utility of Pourbaix diagrams in the context of e‐waste oxidative leaching of metals is also limited because they become intricate for multicomponent systems. The reaction thermodynamics and kinetics of these systems can change dramatically, making predictions very difficult. Factors such as the presence of complexing agents in solution, corrosion of alloys, and non‐alloyed metal mixtures further complicate the determination of multiple‐phase equilibria.^[^ ^147^ ^]^

Methods detailed in Table 1 for leaching noble metals also effectively leach base metals.^[^ ^148^ ^]^ Weak, dilute acids can successfully leach metals like Al, Sn, Zn, and Fe, but fail to leach Cu and Ag due to their positive electrochemical potentials. Even though the standard reduction potentials for Pb^2+^/Pb and Ni^2+^/Ni are negative, HCl is unsuitable for their leaching because PbCl_2_ is insoluble and Ni's reaction with HCl is slow. Cu and Ag can be leached using concentrated HNO_3_. Since Ag and Pb chlorides are insoluble, they precipitate when aqua regia is used for unselective leaching.^[^ ^125^ ^]^ The standard reduction potential is not the only factor predicting acidic dissolution; for example, Sn should better dissolve in HNO_3_ than HCl due to the former being both acidic and oxidizing; unfortunately the layer of SnO_2_ that forms on the surface of tin passivate it.^[^ ^125a^ ^]^

To move toward green hydrometallurgy, it is crucial to minimize the entropy involved in the process by avoiding non‐discriminatory multi‐metal dissolution. This requires a sequential oxidative leaching process that starts with base metals and progresses to noble metals. Using milder and less toxic reagents is essential to respect this oxidative dissolution hierarchy.

For instance, the selective leaching of Sn and Pb from solderings in Waste PCBs (WPCBs) begins with a treatment using 0.2 M HNO_3_, which leaches 99.99% of Pb but not Sn due to passivation. The subsequent step with 3.5 M HCl dissolves the SnO_2_ passivation layer, allowing Sn leaching.^[^ ^149^ ^]^ However, this method does not recover other metals. Simple acid leaching of base metals is highly caustic and may pose safety issues due to hydrogen evolution. Somasundaram et al. (2014) used a two‐step approach to leach base metals from WPCBs: in the first step 92% of Sn was selectively leached, along with trace amounts of Cu, Ni, and Pb using 3 M HCl and 0.1 M CuCl_2_ at 35 °C; the residue of the first stage was treated with 0.5 M CuCl_2_ and 3.0 M HCl resulting in the dissolution at 50 °C of the remaining Cu, Ni, and Pb.^[^ ^150^ ^]^ They did not address Au recovery.

Selective oxidative extraction of base metals from e‐waste using H_2_O_2_ as an oxidizing agent is assisted by chelating agents like diethylene triamine pentaacetic acid (DTPA).^[^ ^151^ ^]^ Quinet developed a sequential method to recover base and noble metals from WPCBs but selectivity was only partial.^[^ ^152^ ^]^

Birloaga and Vegliò (2016) similarly employed a two‐stage leaching process from WPCBs.^[^ ^153^ ^]^

There is a trend toward replacing fossil‐based reagents with biomass‐derived chemicals due to their worker safety, lower environmental impact, high biodegradability, lower volatile organic compounds (VOCs) release, green photosynthetic origin, and reduced disposal costs^[^ ^154^ ^]^

Organic acids, tested for their lower GHG emissions compared to inorganic acids, prove to be a sustainable alternative.^[^ ^155^ ^]^

Chemical pretreatment of pulverized PCBs from cell phones with citrate/ammonium phosphate/H_2_O_2_ solution leached base metals (97% Cu, >80% Fe, Ni, and Zn, and 21% Al), while H_2_SO_4_/H_2_O_2_ solution improved extraction of Cu, Fe, Ni (>90%), and Al (79%). After sulfuric pretreatment, Au was leached using thiourea, and chemical inhibitors (oxalate, thiocyanate) were used to prevent interference from residual base metals, ensuring Au selectivity.^[^ ^151^ ^]^

A four‐step staggered leaching process of coinage metals (Cu, Ag, Au) involved milling e‐waste with Carbsyn, a blend of perfluoro components. Coinage metals, known for their use in making coins due to their durability, conductivity, and resistance to corrosion, are valuable components of electronic waste. The steps included (i) refluxing at high temperatures with citric acid to leach base metals, (ii) reacting the residue with NH_3_/(NH_4_)_2_SO_4_/I_2_/NaOH to selectively leach Cu (recovered via zinc cementation), (iii) leaching Ag at room temperature with Na_2_S_2_O_3_ (recovered by electrowinning), and (iv)leaching Au with I_2_/KI. Gold was recovered by electrowinning, that is the electrodeposition and concomitant reduction of the metal cation at a cathode. Recovery yields for Cu, Ag, and Au from RAM boards were 70%, 92%, and 64%, respectively, though the zinc ions replaced copper ions, reducing the resource efficiency of the process.^[^ ^156^ ^]^

Sequential leaching of coinage metals from pure metal wires using a DES made of choline chloride and urea specifically leached Cu. Density Functional Theory (DFT) calculations predicted the oxidative dissolution of Cu, Ag, and Au, but undesirable oxidation of Ag to AgCl was observed. The authors recommend a fine‐tuning of reaction times and reagent concentrations for multi‐metal substrates to maintain selectivity. Subsequently, Ag was dissolved in lactic acid/H_2_O_2_, and Au was dissolved in choline chloride/urea/oxone (2KHSO_5_ ⋅ KHSO_4_ ⋅ K_2_SO_4_), though efficiency was only moderate.^[^ ^157^ ^]^

In a counterintuitive approach, a mixture of N‐bromosuccinimide and pyridine selectively oxidized Au more than base metals.^[^ ^148^ ^]^

### 4.3 Green Hydrometallurgy

A paradigm shift toward green hydrometallurgy is an ongoing process.^[^ ^33^ ^]^ The standard hydrometallurgical route is time‐consuming, labor‐intensive, and chemically illogical for several reasons: i) it redundantly oxidizes both precious and base metals before selectively reducing them, ii) strong leaching agents harm workers and the environment and is incompatible with needed selectivity, and iii) the process leads to downcycling of precious metals, reducing their purity.

A green strategy involves minimizing entropy investment by avoiding harsh, non‐discriminatory metal dissolution. This reduces or eliminates the need for process purification steps and minimizes energy input. The adoption of a “waste‐for‐waste” approach using biobased, waste‐derived chemicals enhances sustainability.

Green hydrometallurgy, tailored for impurity‐contaminated e‐waste, takes advantage of the chemical architecture of metals in scraps. Gold and other noble metals are usually plated as thin coatings over base metal substrates. Using mild conditions to leach only easily oxidizable base metals releases the gold layer as solid flakes. This allows for the physical separation of metallic gold from the reaction mixture, using simple filtration. This method eliminates the need for crushing and chemical refining, reducing gold loss, saving costs and time, and decreasing environmental pollution. The purity of the recovered goldmatches the original purity, preventing downcycling.^[^ ^33^ ^]^

Despite its benefits, this strategy has been sporadically followed. For example, a hydrochloric acid/iron chloride (1 M each) mixture effectively leached base metals, releasing gold.^[^ ^158^ ^]^ A persulfate solution,^[^ ^159^ ^]^ ammonium persulfate in the presence of O_2_,^[^ ^104^ ^]^ and CuCl_2_/HCl solutions,^[^ ^160^ ^]^ also selectively dissolved base metals, recovering gold as solid particles almost quantitatively.

Methane sulfonic acid with H_2_O_2_ leached copper and nickel under gold plates on mobile phone PCBs, with recovery up to 95% of gold.^[^ ^161^ ^]^ Metal ions like Cu^2+^ or Fe^3+^ and persulfate‐based oxidation, while effective, pose disposal issues due to remaining reaction mixture components.

The sustainability of this rational strategy increases if i) a green oxidant is used for base metal leaching, ii) a biobased weak organic acid provides the needed acidic pH, and iii) the base metal leachate can be used for other productions, avoiding the reductive phase of the hydrometallurgy workflow and enhancing circularity.

The additional advantage is that mild conditions are not harmful and can be achieved using biobased organic acids, which are intrinsically more sustainable.^[^ ^155^ ^]^ Furthermore, the energy consumption required to manufacture an organic acid is significantly lower than that of producing an inorganic acid at the same concentration. This reduction in energy use contributes to a smaller carbon footprint, highlighting the eco‐friendly nature of biobased reagents.

This approach was tested on waste RAM boards^[^ ^33^ ^b]^ using hydrogen peroxide and lactic acid solution to selectively leach only base metals. RAM boards were selected due to their high content of valuable noble metals.^[^ ^162^ ^]^

Hydrogen peroxide is widely used in hydrometallurgical processes because it is a safe, effective, and powerful oxidant (standard potential 1.78 V vs standard hydrogen electrode), stronger than chlorine, chlorine dioxide, and potassium permanganate in acidic solutions.^[^ ^163^ ^]^ Its electrochemical on‐site synthesis with selective electrocatalysts, possibly derived from e‐waste, is energy and resource‐efficient, avoiding the drawbacks of the solvent‐based anthraquinone process.^[^ ^164^ ^]^

The non‐toxicity of the reduction product (water) is noteworthy for its environmental and social sustainability benefits. The acidic environment needed to enhance the reduction potential of H₂O₂ was provided by lactic acid within the “waste‐for‐waste” concept.

Lactic acid is a well‐recognized biobased platform chemical.^[^ ^154^, ^165^ ^]^ Whey, a major pollutant in the dairy industry, is the most suitable raw material for lactic acid production.^[^ ^166^ ^]^

Lactic acid is known to form complexes with metal ions, providing an additional driving force for base metal leaching.^[^ ^167^ ^]^

The presence of copper ions in an acidic environment may trigger a Fenton‐like reaction at room temperature and atmospheric pressures.^[^ ^168^ ^]^

The decomposition of H₂O₂ exhibited pseudo‐first‐order kinetics.^[^ ^168^ ^]^ The Fenton‐active state of copper in its redox cycle is Cu⁺; the Fenton‐like irreversible reaction results in the in‐situ generation of hydroxyl radicals (HO·) and oxygen. Under acidic conditions, the hydroxyl radical, with a standard potential of 2.80 V versus the standard hydrogen electrode, is the main oxidant and significantly contributes to the oxidation of base metals.^[^ ^169^ ^]^ Nickel (II) has also been reported to facilitate the Fenton reaction.^[^ ^168^ ^]^ Heating the mixture to achieve rapid gold peeling in a short time (1 h) is energy‐intensive and hazardous.^[^ ^33^ ^]^

A more effective approach involves allowing complete gold peeling from RAM edges over 12 h at room temperature (21 °C), using minimal reagent concentration and no heating or magnetic stirring, thereby grounding the energy input.^[^ ^33^ ^]^ This method also facilitates the use of the reaction byproduct, copper (II) lactate, for synthesizing the valuable photocatalyst Cu₂O, enhancing circularity.

This strategy has also proven to be successful in recovering palladium^[^ ^33a^ ^]^ and ruthenium^[^ ^33c^ ^]^ from waste copper‐core metal wires used in fashion items, which undergo precious metal plating in galvanic plants.

Solvometallurgy focuses on the recovery of metals using nonaqueous solutions; lately, it is flanking hydrometallurgy. Concerns arise regarding the use of organic solvents. Halogens necessary for the oxidative leaching of metals may dangerously react with these solvents, forming hazardous halogenated compounds.

While solvents can be purified through energy‐intensive distillation, eventually fresh batches of solvent must be introduced, and the spent solvent safely disposed of. Consequently, sustainability concerns arise for solvometallurgical processes due to health, safety, and environmental reasons.^[^ ^170^, ^171^ ^]^

Concerning ionometallurgy, the high cost of ionic liquids (ILs) has limited their use on an industrial scale. A more affordable alternative to ILs is the use of deep‐eutectic solvents (DESs).^[^ ^172^ ^]^ However, their high viscosity can impede mass transport, slow down leaching or electrodeposition kinetics, and complicate solid–liquid separations. These challenges contribute to their relatively low technology readiness level (TRL).

Both ionic liquids and DESs are often deemed green solvents due to their nonvolatility, low flammability, and generally low toxicity. However, their environmental impact can vary significantly depending on their composition.^[^ ^171^, ^173^ ^]^ When considering organic solvometallurgy or ionometallurgy as more sustainable alternatives to hydrometallurgy, it is crucial to ensure that improvements in one phase of the process do not compromise overall sustainability.

### 4.4 Upcycling of Precious Metals Recovered by Green Hydrometallurgy in Optoelectronics

Our research group has advanced beyond traditional green hydrometallurgy for precious metal recovery. We successfully recycled palladium (Pd) to fabricate source and drain electrodes for organic field‐effect transistors (OFETs), which use organic molecular semiconducting thin films as the transistor channel material. Specifically, we utilized phenyl‐C61‐butyric acid methyl ester (PCBM) as the organic semiconductor.^[^ ^33a^ ^]^

**Figure 5:**
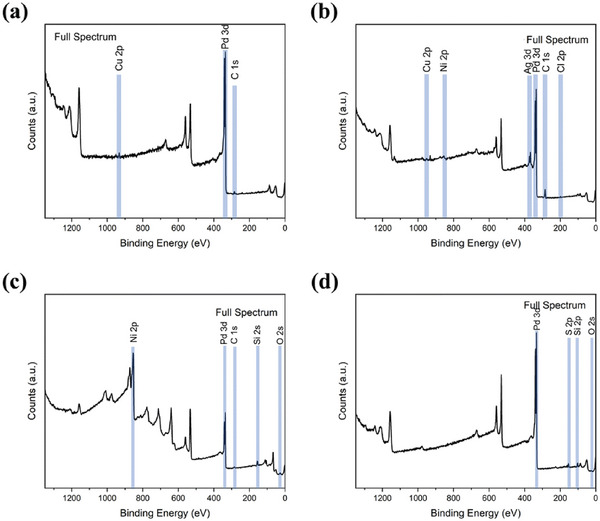
XPS full survey spectra of waste metal wires (a), peeled flakes (b), e‐beam evaporated electrodes from recycled Pd (c), and commercial Pd (d) on SiO_2_.w Adapted with permission.^[^ ^33^ ^] 2023, RCS Publishing.^

Interdigitated circular recycled palladium electrodes, deposited via e‐beam evaporation, were extensively characterized using scanning electron microscopy (SEM), energy‐dispersive spectroscopy (EDS), X‐ray photoelectron spectroscopy (XPS), and atomic force microscopy (AFM) to assess their morphology and surface chemical composition (**Figure** **5**).

Surprisingly, devices with recycled Pd electrodes demonstrated superior performance compared to their commercial Pd counterparts.^[^ ^174^ ^]^ This improvement can be attributed to several factors. First, the recycled Pd electrodes had a greater thickness, which positively influenced performance. Second, these electrodes exhibited lower surface roughness, which facilitated a more uniform coverage by the organic molecular thin film. Additionally, the inclusion of nickel (Ni) in the recycled Pd electrodes, as revealed by EDS and XPS, lowers the Schottky injection barrier at the electrode‐organic semiconductor interface. Specifically, the work function of the Ni–Pd alloy (≈5.04–5.35 eV) is lower than that of pure Pd (≈5.22–5.60 eV), leading to improved electron injection into the PCBM film and higher mobility.^[^ ^33a^ ^]^ Furthermore, the surface of commercial Pd electrodes was more hydrophobic (water contact angle of 90.4 ± 3.5°) compared to the recycled Pd electrodes (water contact angle of 85.6 ± 2.6°).

**Figure 6:**
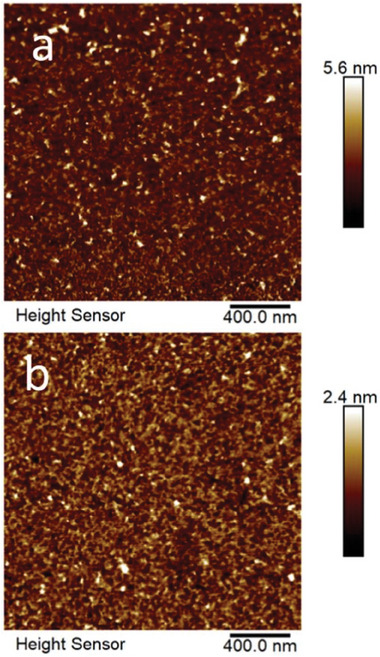
Atomic force microscopy images of e‐beam evaporated Au from (a) commercial and (b) recycled flakes. Adapted with permission.^[^ ^33^ ^]^ 2022, IOP Publishing.

**Figure 7:**
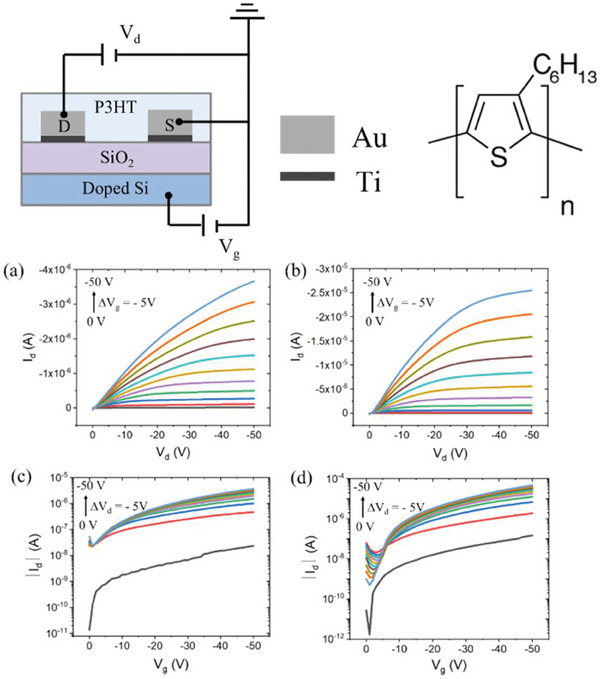
Output (a) and (b) and transfer (c) and (d) characteristics of P3HT field‐effect transistors based on recycled (a) and (c) and commercial (b) and (d) Au source and drain electrodes. The scheme of the P3HT field‐effect transistors and the molecular structure of the repeating unit in P3HT are also shown, respectively on top left and top right of the figure. Adapted with permission.^[^ ^33^ ^]^ 2022, IOP Publishing.

In addition, we investigated the use of recycled gold for the source and drain electrodes in organic transistors based on the polymer poly(3‐hexylthiophene‐2,5‐diyl) (P_3_HT) (**Figure** **6**). The output characteristics showed linearity in the low drain‐source voltage (V_ds_) region, indicating ohmic contact. Both types of transistors had a threshold voltage of ≈−8 V (**Figure** **7**). The ON/OFF ratio (ION/IOFF) was ≈10^3^ for commercial gold and 10^2^ for recycled gold, with hole mobilities of (3.1 ± 0.3) × 10^−4^ cm^2^ V^−1^s^−1^ for commercial gold and (6.3 ± 0.8) × 10^−5^ cm^2^ V^−1^s^−1^ for recycled gold under saturation conditions. In linear conditions, the mobilities were (1.4 ± 0.1) × 10^−4^ cm^2^ V^−1^s^−1^ for commercial gold and (4.9 ± 1.2) × 10^−5^ cm^2^ V^−1^s^−1^ for recycled gold. The increased resistance of recycled gold electrodes in P_3_HT transistors suggests that the recovery process needs optimization to enhance gold purity and, consequently, device performance.^[^ ^33b^ ^]^

#### 4.4.1 Perspective on Valorization Optoelectronic Devices

We are currently investigating the application of our recycled palladium (Pd) electrodes to produce palladium hydride (PdH_x_) protodes for measuring proton conductivity in hydrated biomolecular thin films, aiming to advance sustainable organic bioelectronics.^[^ ^175^ ^]^ Additionally, we are expanding our research to include other precious metals, such as ruthenium from fashion waste and platinum from spent fuel cells. Given its critical role in catalysis, the recycling of ruthenium is essential for enhancing the sustainability of catalytic processes. Furthermore, we plan to utilize copper lactate to synthesize copper oxides, which are promising semiconducting photocatalysts for applications in solar water splitting and nitrogen reduction.^[^ ^33^ ^]^

## 5 Conclusion

The valorization of municipal solid waste presents a sustainable pathway to address the growing challenges of waste management while simultaneously contributing to the advancement of functional materials. This Perspective underscores the potential of converting organic, plastic, and electronic wastes into valuable resources such as functional carbon materials, MOFs and optoelectronic devices based on recovered metals. These advancements have far‐reaching implications for energy conversion, storage, and environmental applications, aligning with the principles of green chemistry and the United Nations' Sustainable Development Goals.

The treatment and valorization of organic waste through various processes, such as anaerobic digestion, gasification, pyrolysis, torrefaction, hydrothermal, and ionothermal carbonization, offer significant opportunities for sustainable energy conversion and the development of value‐added materials. Each process has unique advantages, making them suitable for different types of organic waste and desired outcomes. Microwave‐assisted pyrolysis (MWP) and solar‐assisted pyrolysis stand out as particularly important processes for organic waste valorization due to their unique advantages over traditional methods. In comparison to other organic waste valorization processes, MWP and solar‐assisted pyrolysis offer distinct advantages in terms of energy efficiency, environmental impact, and sustainability. These processes represent the future of organic waste management, with the potential to significantly reduce the carbon footprint and contribute to a circular economy, despite some challenges in terms of cost and reactor design.

Among the various approaches used in converting plastic and electronic waste into MOF, mechanochemical milling stands out due to its environmental and economic advantages, eliminating the need for solvents and operating under ambient conditions, making it scalable and cost‐effective for large‐scale processing. However, challenges such as optimizing milling conditions, ensuring purity and quality control of MOFs, and balancing mechanical energy input and equipment costs against environmental benefits need to be addressed. While solvothermal and hydrothermal synthesis methods can produce high‐quality MOFs, they pose environmental and safety concerns due to the use of high temperatures, pressures, and toxic solvents. Direct synthesis from waste aligns with circular economy principles but faces challenges in precursor purity and extraction optimization. On the converse, the low selectivity (high affinity) for metals observed during the synthesis of MOFs from waste materials turns out to be an advantage in adsorption‐based recovery of precious metals using MOFs. Metal recapture is efficient, despite still requiring further research on scalability and regeneration. To fully evaluate the potential of MOFs, it is crucial to develop standardized protocols for scalable production, advanced purification techniques, and economic strategies to reduce production costs. Addressing these challenges will be key to harnessing MOFs' potential in revolutionizing e‐waste recycling and metal recovery, contributing to a sustainable future for e‐waste management.

The exploration of green hydrometallurgy routes aims to upturn the current hazardous hydrometallurgical approaches, avoiding precious metals oxidation in the leaching step, thereby minimizing the entropy and energy investment. A mild reactive environment, comprising food waste‐based reagents, further exemplifies the importance of developing environmentally friendly processes that recover precious metals in their pristine state, without downcycling. This strategy supports a circular economy approach in which different waste streams can profitably interlock. The reclaimed precious metals were used to fabricate optoelectronic devices, paving the way toward green and sustainable electronics.

## Conflict of Interest

The authors declare no conflict of interest.
